# Advance of Thrombolysis and Thrombectomy in Acute Ischemic Stroke

**DOI:** 10.3390/jcm12020720

**Published:** 2023-01-16

**Authors:** Hyo Suk Nam, Byung Moon Kim

**Affiliations:** 1 Department of Neurology, Yonsei University College of Medicine, Seoul 03722, Republic of Korea; 2 Department of Radiology, Yonsei University College of Medicine, Seoul 03722, Republic of Korea

Globally, stroke remains the second leading cause of death, and the third-leading cause of death and disability, in the world. Stroke burden increased substantially [1]. The use of intravenous recombinant tissue plasminogen activator (IVT) has been the standard of care for treating acute ischemic stroke for over two decades; however, its low efficacy and potential harmfulness limit its effectiveness. After successful randomized clinical trials, endovascular thrombectomy (EVT) has become the mainstay treatment of large vessel occlusion in patients with acute ischemic stroke. However, the guidelines still recommend that patients eligible for IVT should receive it. Two randomized clinical trials showed that EVT was effective even in the late time window of up to 16 or 24 h [2,3]. Many researchers have conducted research into more effective treatments, evaluation tools, and efficient care pathways in thrombolysis and thrombectomy in patients with acute ischemic stroke.

The recanalization rate of EVT is about 70–80% and the number needed to treat is 2.6 with a low complication rate. However, more than half of patients remain functionally dependent 3 months after the initial stroke. It remains unclear whether EVT will be beneficial in all stroke patients. Recanalization is one of the critical factors affecting early and late outcomes following acute ischemic stroke. Kim et al., investigated whether stroke risk scores are associated with unsuccessful recanalization in patients who received EVT [4]. They tested the CHADS_2_, CHA_2_DS_2_-VASc, ATRIA, and Essen stroke risk scores, and found that all stroke risk scores were associated with recanalization failure after EVT. This study showed the association between stroke risk scores and the clinical outcomes of patients who received EVT.

Successful recanalization does not always translate into good clinical outcomes. When successful recanalization fails to bring a favorable prognosis, it is called futile recanalization (FR). FR is usually defined as the modified Rankin scale ≥3 at 3 months despite successful recanalization (Thrombolysis in Cerebral Infarction grade ≥ 2b). The prevalence of FR has been reported to range from 29 to 67%. Causes of FR can be divided into baseline patient characteristics, imaging findings, and postprocedural factors. The baseline characteristics of old age, female gender, higher initial National Institutes of Health Stroke Scale score, comorbidities, systolic blood pressure, glucose and biomarkers, and late treatment are reported. Imaging factors included large infarction, poor collaterals, proximal occlusion, and white matter hyperintensity. Procedural factors included no IVT use, symptomatic intracranial hemorrhage, blood pressure variability, and blood pressure controls after recanalization.

EVT in older people can be challenging, as older people often live with frailty, which may be associated with FR and poor outcomes. Schnieder et al., investigated whether frail patients also face adverse outcomes after EVT [5]. They found that frailty led to poor functional outcomes and higher mortality in patients undergoing EVT. Krajíčková et al., studied real-world data of elderly patients who were treated with EVT [6]. They found that patients ≥ 80 years old and undergoing IVT were less likely to achieve a 3-month good clinical outcome. Women were highly associated with 3-month good clinical outcomes and lower 3-month mortality.

The prediction of prognosis based on advanced imaging techniques before IVT or EVT is promising. Incorporating advanced neuroimaging in patients with acute ischemic stroke may increase the yield of IVT administration without affecting the treatment’s effectiveness and safety [7]. Among various tools, hypoperfusion severity can be estimated using the hypoperfusion intensity ratio (HIR). Baek et al., studied the association between HIR and clinical outcomes [8]. They found that the low-HIR group had a more favorable outcome, even with an unfavorable Alberta Stroke Program Early CT score and onset-to-recanalization time. Using pre-treatment CT angiography, HIR provides an objective numerical value compared to classic tools that provide a qualitative or merely categorical value. Kim et al., demonstrated the feasibility of a machine-learning-based tissue outcome prediction technique using features derived from pre-treatment, perfusion-weighted, and diffusion-weighted imaging [9].

During and after thrombolysis and thrombectomy, contrast-associated acute kidney injury can deteriorate patients. Yoo et al., studied the retrospective cohort of three stroke centers in Korea, finding that AKI occurred in 9.8% of EVT patients [10]. The occurrence of AKI was associated with a poor functional outcome and mortality at 3 months. Accompanying coronavirus disease 2019 (COVID-19) in patients with acute ischemic stroke is problematic. However, safety and efficacy data of IVT and EVT in patients with COVID-19 are scarce. Jurkevičienė et al., found that reperfusion therapies in COVID-19 patients are safe. Functional outcomes at 3 months were significantly worse and 3-month mortality was higher than the control group [11].

Until the early 1990s, managing patients with stroke had long remained a conservative treatment. After the US FDA approved recombinant tissue plasminogen activator in 1995, stroke management was never the same. Likewise, the current method of EVT is a mainstay in patients with acute large vessel occlusion. The development of treatment tools and sophisticated care after reperfusion therapy improves outcomes. As promising as the future looks, we will meet the next generation of reperfusion therapy and care.
